# The thyroid-ovary axis: beyond coincidental endocrine similarities

**DOI:** 10.1590/1806-9282.20260916

**Published:** 2026-11-02

**Authors:** Gulru Birce Sonmezoz, Evrim Cakir

**Affiliations:** 1 Taksim Training and Research Hospital, Department of Internal Medicine – Istanbul, Turkey.; 2 Taksim Training and Research Hospital, Department of Endocrinology and Metabolic Diseases – Istanbul, Turkey.

Dear Editor,

Although the thyroid gland and ovary are traditionally considered distinct endocrine organs, accumulating evidence suggests that they share structural, hormonal, and functional characteristics. Recognition of these similarities offers a useful perspective for understanding several clinical observations involving both thyroid and reproductive health.

A notable structural similarity between the thyroid gland and ovary is the presence of a predominantly simple cuboidal epithelium. However, the ovarian surface epithelium ranges from squamous to cuboidal, whereas the thyroid follicular epithelium ranges from squamous to columnar depending on functional activity^1,2^.

Beyond this histological resemblance, both tissues demonstrate responsiveness to common endocrine signals. Human ovarian tissue, including oocytes, granulosa cells, stromal cells, and surface epithelial cells, has been shown to express thyroid-stimulating hormone (TSH) receptors, while thyroid hormone receptors are expressed in granulosa cells, cumulus cells, and oocytes^3,4^. Furthermore, thyroid hormones are detectable within follicular fluid, suggesting a direct role in ovarian physiology^5^. An experimental study has demonstrated that triiodothyronine (T3) influences gene expression associated with inflammation and estrogen receptor (ER) α expression in ovarian surface epithelial cells^6^, suggesting that thyroid hormones may actively participate in ovarian cellular regulation rather than exerting solely indirect systemic effects. An association between ovarian cancer and hyperthyroidism has been reported in some studies, although the underlying mechanisms remain unclear and may involve shared hormonal signaling pathways^6,7^.

On the other hand, thyroid tissue is responsive to reproductive hormones. Estrogen and progesterone modulate thyroid follicular cell activity^8,9^, and both estrogen and progesterone receptors are frequently expressed in papillary thyroid carcinoma^10^. Estradiol has been shown to stimulate thyroid cell proliferation and may contribute to thyroid growth under certain physiological and pathological conditions, including papillary thyroid cancer^11,12^. Interestingly, oral contraceptive use has been associated with reduced thyroid volume and a lower prevalence of goiter^13^. These apparently divergent findings may also reflect differences in the type, dose, and route of administration of estrogenic compounds^14^. An experimental animal study has demonstrated that conjugated equine estrogens induce a hyperstimulatory effect on thyroid morphology, characterized by reduced follicular and colloid areas, increased epithelial height, and enhanced proliferative activity, while tamoxifen exerted distinct histomorphological effects, suggesting that the structural impact of estrogenic compounds on the thyroid is compound-specific^15^.

While increased thyroid volume has been reported in women with polycystic ovary syndrome (PCOS), which is characterized by hormonal fluctuations, exogenous estrogen therapy is typically administered in a more stable hormonal context. This suggests that endogenous and exogenous hormonal exposures may exert distinct biological effects. In particular, continuous exogenous estrogen therapy is associated with suppression of luteinizing hormone (LH), which has been proposed to have independent effects on thyroid physiology. Therefore, differences in thyroid outcomes between these states likely reflect variations in overall endocrine feedback regulation and gonadotropin dynamics rather than estradiol exposure alone. Consistent with this interpretation, a large prospective study in women with hysterectomy alone found no association between hormone replacement therapy and thyroid cancer risk^16^.

The existence of struma ovarii further illustrates the biological overlap between these organs. As a specialized ovarian teratoma predominantly composed of thyroid tissue, struma ovarii demonstrates that thyroid tissue can develop and remain functionally active within the ovary. The biochemical manifestations of hyperthyroidism associated with struma ovarii closely resemble those observed in primary hyperthyroidism^17^.

Shared hormonal signaling pathways provide additional evidence for this relationship. TSH, follicle-stimulating hormone (FSH), and LH are structurally similar heterodimeric glycoproteins and consist of an identical α-subunit with structurally distinct β-subunits^18^. FSH has been shown in experimental animal models to induce thyroid follicular changes resembling those observed following TSH stimulation^19^. Experimental studies have demonstrated that LH can stimulate thyroid adenylate cyclase activity and enhance iodine uptake by thyroid tissue^20,21^. Consistent with these findings, clinical studies have identified a positive association between thyroid volume and LH levels in women of reproductive age^22^. In contrast, the use of oral contraceptives was associated with a decreased thyroid volume and a fourfold reduction in the risk of goiter^13^. The distinct effects between endogenous estradiol fluctuation and exogenous estrogen exposure may be partly attributable to differences in LH regulation.

The relationship becomes particularly evident during pregnancy through the actions of human chorionic gonadotropin (hCG), which shares a common α-subunit with TSH, FSH, and LH. It has been shown that hCG can stimulate thyroid activity in a manner similar to TSH, leading to increased TSH receptor expression, iodine uptake, adenylate cyclase activity, and thyroid hormone secretion^22^. During early pregnancy, elevated hCG levels exert a transient thyrotropic effect via the TSH receptor, leading to stimulation of thyroid growth, increased production of T4 and T3, and subsequent suppression of TSH^23^. This interaction may represent a physiological maternal adaptation that supports increased thyroid hormone availability during early pregnancy.

Another clinically relevant link between the thyroid and ovary is the association between autoimmune thyroid disease and premature ovarian insufficiency. Autoimmune thyroiditis has been identified in approximately 14–32.7% of women with premature ovarian insufficiency at the time of diagnosis^24^. However, current evidence suggests that thyroid autoantibody positivity alone is not consistently associated with diminished ovarian reserve, indicating that accompanying thyroid dysfunction, rather than thyroid autoimmunity itself, may play a more important role in reproductive impairment^25^.

Clinical studies also suggest that thyroid-directed therapies may directly affect ovarian function. Radioactive iodine (RAI) treatment has been associated with menstrual irregularities and reductions in anti-Müllerian hormone (AMH) levels, indicating a potential adverse impact on ovarian reserve^26,27^. While current evidence does not establish a direct causal relationship between all thyroid interventions and impaired fertility, these findings emphasize the importance of considering reproductive outcomes when selecting treatment strategies for women of reproductive age. They also highlight the need for prospective studies evaluating the reproductive safety of emerging thyroid therapies, such as radiofrequency and microwave ablation.

In conclusion, available evidence suggests that the thyroid and ovary share more than a coincidental endocrine relationship. Structural similarities, common receptor expression patterns, overlapping hormonal signaling pathways, and shared clinical phenomena collectively support the existence of meaningful biological interactions between these organs. Although many observations remain associative and mechanistic studies are still limited, viewing thyroid–ovarian interactions within an integrated endocrine framework may provide new insights into reproductive disorders, thyroid disease, and treatment-related complications. Future research should aim to further elucidate the molecular mechanisms underlying thyroid–ovary interactions and evaluate the reproductive consequences of thyroid diseases and their treatment.

## Data Availability

Data sharing is not applicable to this article as no new data were created or analyzed in this study.
